# Spondylocostal Dysostosis: A Literature Review and Case Report with Long-Term Follow-Up of a Conservatively Managed Patient

**DOI:** 10.1155/2018/1795083

**Published:** 2018-03-22

**Authors:** Brendan R. Southam, Adam P. Schumaier, Alvin H. Crawford

**Affiliations:** ^1^Department of Orthopaedics and Sports Medicine, University of Cincinnati, Cincinnati, OH, USA; ^2^Cincinnati Children's Hospital, Cincinnati, OH, USA

## Abstract

**Introduction:**

Patients with spondylocostal dysostosis (SCD) have congenital spine and rib deformities associated with frequently severe thoracic insufficiency and respiratory compromise. The literature is largely composed of case reports and small cohorts, and there is little information regarding adults with this condition. In this report, we describe the natural history of a conservatively treated patient and include quality-of-life issues such as childbearing, athletic participation, and occupational selection.

**Case Presentation:**

We present a patient with SCD who was conservatively treated by a single physician from birth for 31 years. Our patient was capable of a reasonably good quality of life through adulthood, including participation in gymnastics and employment. At age 18, she became pregnant and subsequently terminated the pregnancy due to obstetrical concerns for compromised respiration. She has had intermittent respiratory complaints and occasionally experiences dyspnea with exertion, but this only has limited her during certain activities in the past three years. Currently, she takes naproxen for chronic back pain with periodic exacerbations.

**Discussion:**

Other cases in the literature have described adult SCD patients who have received nonoperative treatment and achieved a wide range of functional outcomes. This provides some limited evidence to suggest that select patients with SCD may be treated conservatively and achieve a reasonable quality of life. However, close clinical follow-up with these patients is recommended, particularly early on, considering the high rates of infant morbidity and mortality. Chest physiotherapy and early pulmonary care have been associated with favorable outcomes in infancy. Surgery to increase thoracic volume and correct scoliosis has been shown in some cases to improve respiratory function. Treatment depends on the degree of thoracic insufficiency and quality of life. The natural history of SCD remains largely unknown, but some patients are capable of relatively favorable life spans, employment, and participation in athletics.

## 1. Introduction

In 1938, Jarcho and Levin described two siblings who were noted to have short necks and trunks due to vertebral segmentation defects and rib anomalies [1]. Throughout most of the 20th century, the term Jarcho-Levin syndrome (JLS) was used to describe two disorders affecting the axial skeleton which are now recognized as distinct entities [2], spondylocostal dysostosis (SCD) and spondylothoracic dysplasia (STD). Both disorders have associated rib malformations and vertebral segmentation defects resulting in some degree of kyphoscoliosis, with each disorder having its own distinguishable radiographic appearance. Notably, in SCD the ribs are asymmetrically fused, missing, or overgrown, whereas in STD the ribs are intrinsically fairly normal, but they are symmetrically fused posteriorly at the costovertebral joint resulting in a crab-like or fan-shaped appearance [3, 4]. The focus of this report is on SCD.

Spondylocostal dysostosis has been associated with significant infant mortality and poor outcomes resulting from thoracic insufficiency and subsequent respiratory complications [5]. Given the relative rarity of this disorder, the natural history of these patients remains largely unknown. An SCD English literature search revealed that only 13 publications mentioned adult patients [6–18]; few had thorough clinical descriptions and just 3 were published in orthopaedic journals [10, 13, 15]. The longest continuous follow-up of a patient with SCD is 23 years [14]. In this report, we describe the relatively favorable 31-year clinical history of a patient with spondylocostal dyostosis, Klippel–Feil anomaly (KFA), and type II sacral agenesis. The patient was seen in the newborn nursery, followed, and treated continuously by a single orthopaedic surgeon.

## 2. Case Presentation

### 2.1. Birth

Patient AM is a Caucasian female born at a community hospital by cesarean section to a 20-year-old gravida 1 para 0 mother of Western European descent. The mother had regular prenatal care, took prenatal vitamins and did not use alcohol, tobacco, or illicit drugs during the pregnancy. During prenatal visits, the fetus had reassuring vitals so an ultrasound was not obtained. The pregnancy was complicated by gestational diabetes and preeclampsia for which the mother had been placed on bedrest during the final trimester. At 42 weeks gestation, the mother was induced, but during labor, fetal distress was noted, requiring an emergency cesarean section to be performed. Birth weight was 7 lbs 4 oz. (3.29 kg) and length was 17″ (43.18 cm). After birth, our patient failed to spontaneously void and concern for acute renal failure resulted in her being transferred to a tertiary pediatric hospital for further evaluation. This later resolved during the hospitalization.

**Figure 1:**
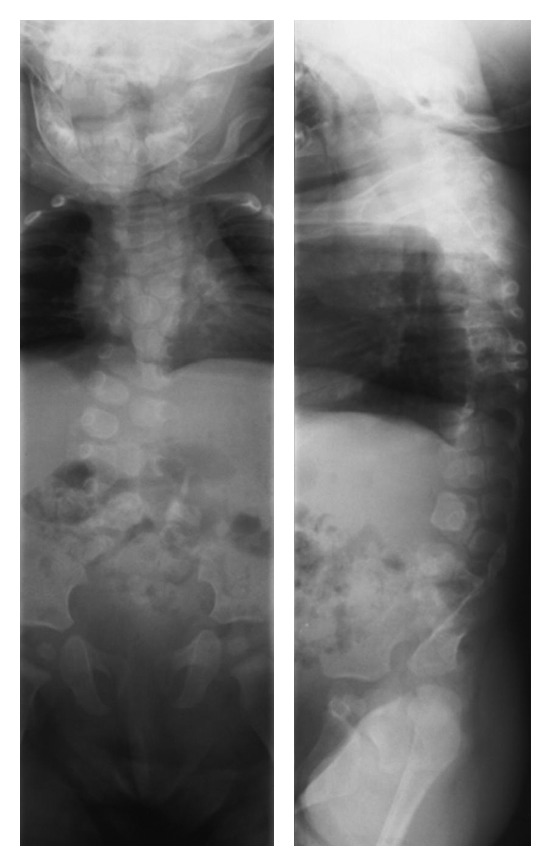
Anteroposterior (AP) and lateral radiographs obtained at 6 months of age demonstrating multiple, asymmetric vertebral segmentation defects of the thoracolumbar vertebra accompanied by asymmetric rib deformities, partial distal sacral agenesis, and fused cervical vertebrae.

During the admission, radiographs of the chest, abdomen, and pelvis were obtained. The images demonstrated multiple vertebral segmentation defects in the thoracolumbar spine and agenesis of the distal sacrum and coccyx. Our patient was noted to have fusion of multiple vertebrae within the cervical spine consistent with a Klippel–Feil deformity and partial fusion of multiple ribs bilaterally consistent with spondylocostal dyostosis. She was referred to pediatric orthopaedics. Since our patient was healthy and without other congenital anomalies, she was conservatively followed with serial radiographic evaluations every 6 months (Figure 1). On exam, she was noted to have a short neck with limited range of motion. Bowel and bladder function were normal. Motor responses were good, and there was full strength in the lower extremities.

### 2.2. Infancy through Adolescence

She began walking at eight months old. At one year of age, she was noted to have a 40° thoracolumbar scoliosis. At age three, she was noted to have a disproportionately short trunk, but was otherwise well developed and wanted to participate in gymnastics. Over the next few years, our patient's congenital spinal deformity was stable. By age seven, she had developed an extremely protuberant abdomen and had prominences of her rib cage due to relatively little longitudinal growth of the spine. Her 3-year-old younger sister, who had no evidence of skeletal deformity, had surpassed her in height at this point. However, the patient was otherwise asymptomatic and doing well in school, subsequently participating in gymnastics, tumbling, and cheerleading with only minimal episodic back pain.

At age ten, radiographs began to demonstrate evidence of consolidation in the thoracic spine. Our patient continued to remain very active over the ensuing years with cheerleading and dance but had increasing back and chest wall pain with these activities. Due to her short truncal stature, she also began to have pain related to costo-iliac impingement that occurred when she bent over, sat, or coughed. She was managed conservatively with NSAIDs and core strengthening exercises which provided some relief. Notably, the patient did not have shortness of breath with her regular activities or frequent respiratory illnesses. By age 14, her spine had almost completely fused. She continued to follow-up with her orthopaedic surgeon every 3 years and had no notable progression of her spinal deformity.

### 2.3. Adulthood

At the age of 18, she became pregnant but subsequently terminated the pregnancy due to obstetrical concerns for compromised respiration secondary to limited thoracic volume. At the time, she also underwent a tubal ligation to prevent future pregnancies. At 21, she returned for follow-up. She was working a job which required her to remain standing throughout her shift that was causing her to experience increasing hip and knee pain. She also noted increasing back pain without neurological deficits that had progressed over the last 3 years. She had a notable leg length discrepancy due to pelvic obliquity, a result of her scoliosis. She was treated with a 5/8-inch shoe lift which provided some relief.

**Figure 2:**
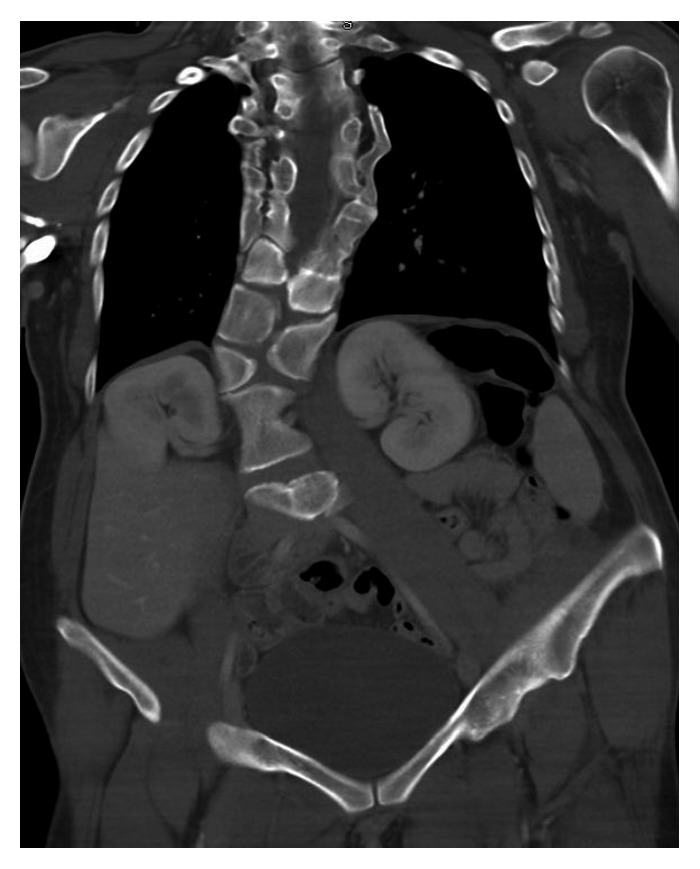
Coronal computed tomography (CT) scan of the chest and abdomen taken at 28 years of age illustrating the vertebral segmentation defects and a stable scoliotic curve. What appeared to be disc spacing has mostly undergone segmental fusions.

**Figure 3:**
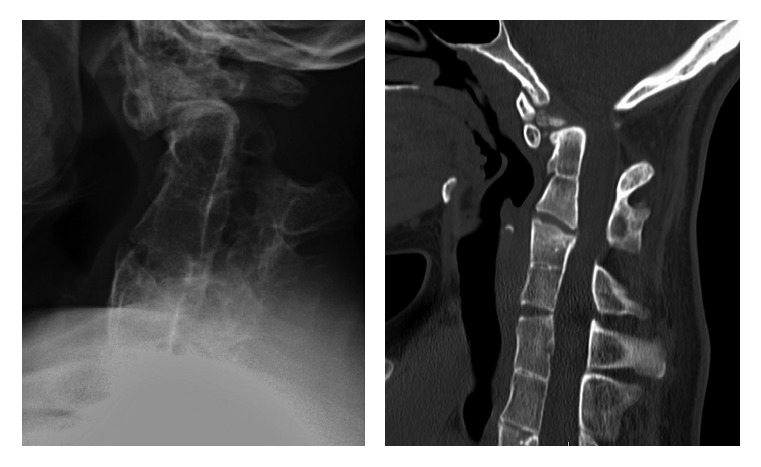
Lateral cervical spine and sagittal CT scan of the cervical vertebrae showing fusion of C2-C3, C4-C5, and C6-C7.

**Figure 4:**
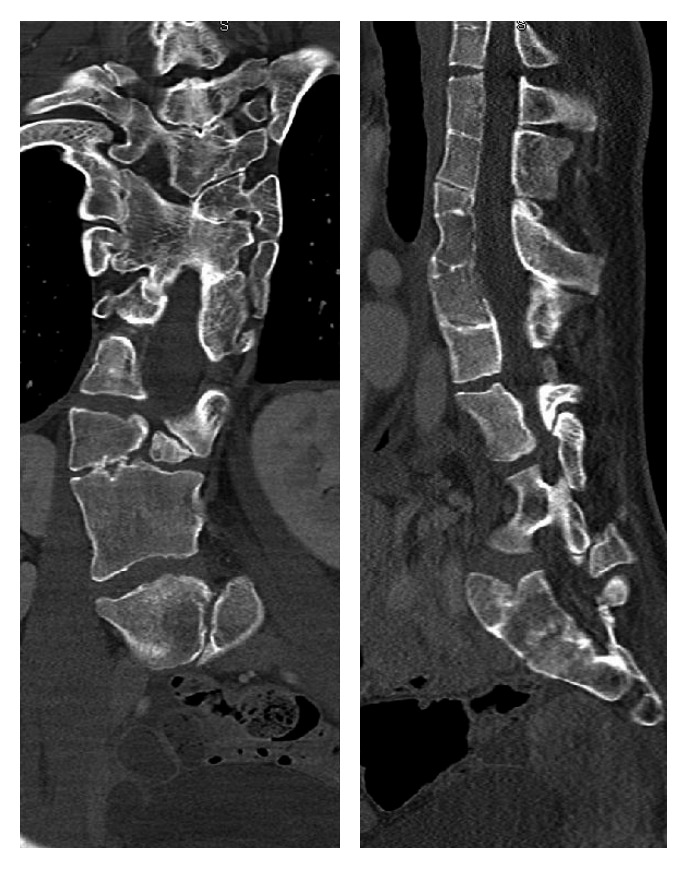
Coronal and sagittal CT scan of the thoracolumbar spine and sacrum demonstrating a variety of vertebral segmentation defects including hemi-, wedge-, butterfly-, and block vertebra.

**Figure 5:**
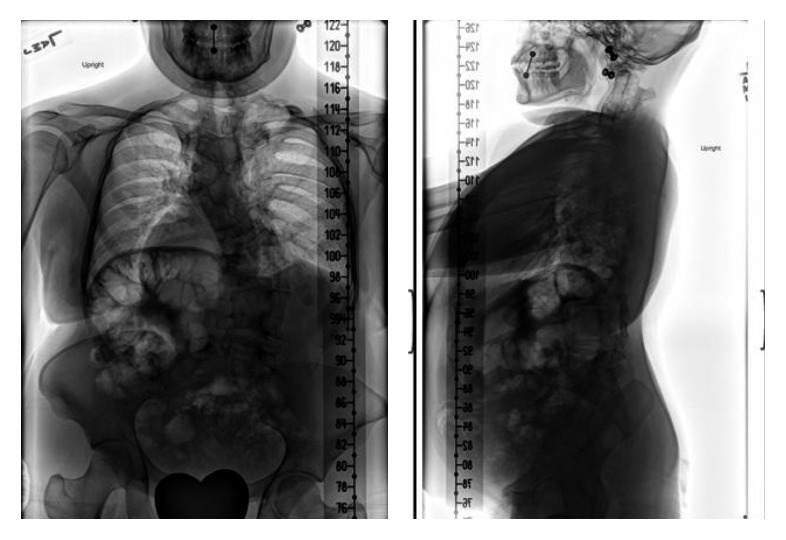
PA and lateral chest radiograph illustrating the asymmetric rib deformities including posteriorly fused and malformed ribs.

At the age of 25, she experienced an episode of severe back pain for which she visited the emergency department (ED). Radiographs were obtained which could not definitively rule out a vertebral compression fracture, so an outpatient bone scan was obtained. The scan demonstrated multiple foci of increased uptake in the vertebral bodies of the cervical, thoracic, and lumbar spine as well as the costovertebral region. These nonspecific findings were attributed to stress reactions from the patient's multiple vertebral anomalies. Several years later, she was involved in a high-speed MVA and was transported to the ED complaining of significant neck and back pain. At the time, computed tomography (CT) of the chest, abdomen, and pelvis and a posteroanterior (PA) chest film were obtained which illustrated her known vertebral, costal, and pelvic anomalies with no evidence of acute fractures (Figures 2–4). She was next seen at age 30 after an accident on a “slip and slide” in which she dove and noted immediate pain in the lower right rib cage. Radiographs illustrated a nondisplaced fracture of the right 7th rib near the costochondral junction which was managed conservatively with NSAIDs (Figure 5). The fracture subsequently went on to heal with resolution of symptoms at one month.

She is now 31 years of age and currently suffers from chronic back pain for which she takes naproxen daily. Two to three times per year she has exacerbations of mid-back pain, necessitating a visit to the emergency department for intravenous narcotic analgesics. She also complains of intermittent sciatic neurogenic symptoms including numbness and paraesthesias down the right lower extremity which are exacerbated by sitting in the car for extended periods of time. She occasionally has dyspnea with exertion after walking long distances or climbing the stairs. She feels that her shortness of breath has always been present, but only has limited her during certain activities in the past three years. Given the intermittent nature of her respiratory issues, she has not required a pulmonary function workup; however, this may be necessary moving forward. She currently smokes a half pack of cigarettes daily and has a 3-pack year history. She is being encouraged to quit. She is now 54 inches tall (4′6″) and weighs 138 pounds. She has been counseled on diet and exercise given the risk of abdominal obesity causing further restriction of lung volume. She completed a Scoliosis Research Society 30 Questionnaire (SRS 30) at her most recent follow-up and 5 years prior. Notably, she felt that she looked worse in clothes and had a more negative body image at present. However, she has increased her level of activity despite increasing back pain at rest.

## 3. Discussion

With genetic studies, case reports, and prospective clinical trials, our understanding of SCD has improved greatly since it was first described. Clinically, the diagnosis of SCD is suggested by a short webbed neck, short trunk, protuberant abdomen, and scoliosis. Individuals can have a number of cooccurring defects including urogenital and anal anomalies, hernias, heart malformations, neural tube defects, and lower limb defects [2]. Radiographically, patients with SCD will have multiple vertebral segmentation defects including hemi-, butterfly-, absent-, or fused vertebrae. It can be transmitted in both an autosomal recessive and autosomal dominant manner [5].

The field of molecular genetics has significantly improved our knowledge and understanding of this disorder and its variations. This child presented prior to the routine assessment of molecular genetics in this patient group. There was a brief period during her adolescence where she was not seen in clinic, became pregnant, and underwent termination of the pregnancy with tubal ligation. At that time, the senior author did not think molecular genetics would add value to the management and care of this patient. The presence of normal siblings causes us to question the ethical justification and the expense of molecular genetic investigation in this patient. Her abdominal girth and weight gain is her greatest concern, and she has inquired about having gastrointestinal banding.

This case report describes the cooccurrence of multiple disorders related to axial skeleton development: spondylocostal dysostosis, Klippel–Feil anomaly (cervical vertebral fusion), and distal sacral agenesis (caudal regression). Cervical segmentation defects and sacral agenesis have previously been described in SCD patients [5, 19]. The cervical defects in this case report were limited to vertebral fusion which has previously been reported in 10% of patients with SCD [5]. Kaissi et al. [10] also described 2 siblings with SCD who had diffuse cervical fusion to the skull base and clivus.

Our patient had minimal respiratory complaints until recently, but the major cause of morbidity and mortality in SCD is thoracic insufficiency leading to decreased lung volume and underdevelopment [20]. This can cause tachypnea, fatigue, and pulmonary infections. Prospective studies remain limited, but advances in neonatal pulmonary care and surgery may be the reason why infant mortality has decreased. In a study by Teli et al. of 13 patients with SCD [20], prenatal diagnosis, treatment of lower respiratory infections, and early chest physiotherapy (CPT) was successful for 11 infants; however, 2 patients refractory to conservative management required surgery.

The surgical treatments described in the literature for thoracic insufficiency in SCD include vertical expandable prosthetic titanium ribs (VEPTR) and chest wall reconstruction with latissimus dorsi flap transfers or polypropylene mesh. In patients with chest wall defects who have herniation of thoracic contents and paradoxical chest wall movements during respiration, the use of a reinforcing latissimus dorsi flap has been shown to significantly improve respiratory function and decrease infections in small case series [21, 22]; however, latissimus dorsi flaps do not directly address the issue of thoracic insufficiency, which is better managed with VEPTR. The goal of VEPTR is to improve scoliotic curves and expand thoracic volume to create space for lung development. In short, one or more vertically oriented distracting devices are attached superiorly to the ribs and inferiorly to the ribs, spine, or pelvis and serially lengthened as the child grows. The technique has been described in detail by Campbell et al. [23, 24] and multiple studies have demonstrated improved clinical respiratory function in SCD patients [ 24, 25, 26]. In a recent study by Ramirez et al. of VEPTR treatments performed in 20 SCD patients, spinal deformity did not progress in 70% of the patients and improvements in assisted ventilation rates were observed in 5 patients; there was no change in 14 patients and no deaths over the mean 4-year follow-up [26]. In a series by Karlin et al., 10 SCD and 19 STD patients received VEPTR. Improvements were noted in thoracic symmetry, spinal deformity, and respiratory function. However, there was no comparison group in this study [25].

Our patient became pregnant and subsequently terminated the pregnancy due to concerns for respiratory insufficiency. The literature regarding pregnancy in patients with SCD is limited, but Dolak and Tartt [7] described a patient with 1 prior vaginal and 2 prior cesarean deliveries who successfully underwent a 3rd cesarean delivery. It is likely that other adult patients with SCD have successfully undergone childbearing, but due to the relative rarity of these conditions, it has not been reported. Evaluating the safety of pregnancy in SCD patients should involve a careful assessment of the patient's respiratory reserve and involve close communication among the obstetrician, anesthesiologist, and pulmonologist throughout the pregnancy.

We reported a patient with SCD successfully managed with conservative treatment for 31 years. The senior author has practiced pediatric orthopaedics at a major children's hospital for 39 years and is experienced with treating this condition. Our patient was capable of a reasonably good quality of life through adulthood, including participation in athletics and employment. Other cases in the literature have described adult SCD patients who have received nonoperative treatment and achieved a wide range of functional outcomes ranging from asymptomatic to requiring oxygen supplementation. [17, 27] Rimoin et al. describe a mildly limited 34-year-old, [28] Norum and McKusick describe [29] a 45-year-old, and Shimizu et al. describe [27] a 61-year-old with this disorder. Whittock et al. mentioned an 82-year-old with limited spine mobility but no complaints or neurologic symptoms. The patient's two daughters, 40 and 41 years old, had more severe phenotypes; they were limited by back pain and fatigue but were both able to hold employment [18]. This provides some limited evidence to suggest that select patients with SCD may be treated conservatively and achieve reasonable outcomes with significant life spans. However, close clinical follow-up with these patients is recommended, particularly early on in life, considering the high rates of infant mortality observed.

Treatment of SCD depends on the degree of thoracic insufficiency, concurrent pulmonary complications, presence of chest wall defects, and quality of life. The ideal time to operate should maximize growth potential and minimize respiratory morbidity. This timeframe remains undetermined [30]. It is the senior author's contention that what appears to be intervertebral disc spaces on early X-rays are actually cartilaginous growth plates that proceed to closure and fusion over time; therefore, not all curves will progress. Consistent with the literature, if significant decline in function and respiratory status or failure to thrive is observed, then surgical intervention with VEPTR should be considered [25, 31].

In conclusion, SCD is a rare disorder characterized by rib and vertebral abnormalities that may result in significant respiratory compromise. While frequent infant mortality was previously observed, more recent studies are reporting patients reaching adulthood. Techniques for prenatal ultrasound diagnosis [32, 33], improved respiratory care in infancy [20], and surgical correction of thoracic insufficiency [26, 30, 31] are all expected to further improve the prognosis of these individuals. Due to thoracoabdominal constraints, we recommend chemical/surgical sterility or cesarean section in female patients with SCD.
